# Advance Care Planning Experiences Among Sexual and Gender Minority People

**DOI:** 10.1001/jamanetworkopen.2022.22993

**Published:** 2022-07-20

**Authors:** Amanda Jane Reich, Stephen Perez, Julia Fleming, Priscilla Gazarian, Adoma Manful, Keren Ladin, Jennifer Tjia, Robert Semco, Holly Prigerson, Joel S. Weissman, Carey Candrian

**Affiliations:** 1 Center for Surgery and Public Health, Brigham and Women’s Hospital, Boston, Massachusetts; 2 Emory University Rollins School of Public Health, Atlanta, Georgia; 3 Fenway Health, Boston, Massachusetts; 4 University of Massachusetts, Boston; 5 Vanderbilt University, Nashville, Tennessee; 6 Department of Occupational Therapy and Community Health, Tufts University, Medford, Massachusetts; 7 University of Massachusetts School of Medicine, Worcester; 8 Harvard Medical School, Boston, Massachusetts; 9 Center for Research on End-of-Life Care, Weill Cornell School of Medicine, New York City, New York; 10 Division of General Internal Medicine, University of Colorado School of Medicine, Aurora

## Abstract

**Importance:**

Advance care planning (ACP) can promote patient-centered end-of-life (EOL) care and is intended to ensure that medical treatments are aligned with patient’s values. Sexual and gender minority (SGM) people face greater discrimination in health care settings compared with heterosexual, cisgender people, but it is unknown whether such discrimination occurs in ACP and how it might affect the ACP experiences of SGM people.

**Objectives:**

To increase understanding of barriers and facilitators of ACP facing SGM individuals.

**Design, Setting, and Participants:**

This mixed-methods national study of ACP included a telephone survey of self-identified SGM and non-SGM participants in a nationally representative sample drawn from a larger omnibus national panel by SSRS. Qualitative interviews were conducted with a subset of survey participants who identified as SGM. Data were collected from October 2020 to March 2021.

**Exposures:**

Self-identified SGM.

**Main Outcomes and Measures:**

The survey included 4 items from the validated ACP Engagement Survey, adapted to capture experiences of discrimination. Interviews asked about participants’ experiences with ACP, including the appointment of medical decision-makers, sharing preferences, and experiences within the health care system more broadly.

**Results:**

A total of 603 adults participated in the survey, with 201 SGM individuals (mean [SD] age, 45.7 [18.7] years; 101 [50.2%] female; 22 [10.9%] Black, 37 [18.4%] Hispanic, and 140 [69.7%] White individuals) and 402 non-SGM individuals (mean [SD] age, 53.7 [19.2] years; 199 [49.5%] female; 35 [8.7%] Black, 41 [10.2%] Hispanic, and 324 [80.6%] White individuals). Regarding reasons for not completing ACP, SGM respondents, compared with non-SGM respondents, were more likely to say “I don’t see the need” (72 [73.5%] vs 131 [57.2%], *P* = .006) and “I feel discriminated against by others” (12 [12.2%] vs 6 [2.6%], *P* < .001). Of 25 completed interviews among SGM participants, 3 main themes were identified: how fear and experiences of discrimination affect selection of clinicians and whether to disclose SGM identity; concerns about whether EOL preferences and medical decision-makers would be supported; and a preference to discuss EOL decisions and values outside of clinical settings.

**Conclusions and Relevance:**

This study found that fear of disclosing sexual orientation or gender identity information and discrimination are important barriers to ACP for SGM in clinical settings, but discussions of preferences and values still occur between many SGM people and medical decision-makers. More SGM-specific patient-centered care might better support these discussions within the health care system. Furthermore, health systems can facilitate improved engagement by supporting clinician sensitivity training, including guidance on documentation and requirements.

## Key Points

**Question:**

What are the barriers and facilitators to advance care planning discussions for sexual and gender minority (SGM) people?

**Findings:**

In this qualitative study of 201 SGM people and 402 non-SGM people, survey participants reported experiences of discrimination in health care, and interview participants described concerns regarding whether their preferences would be supported.

**Meaning:**

The findings suggest that discrimination in the health care system is an important barrier to advance care planning for SGM people.

## Teaser

This qualitative study aims to increase understanding of barriers and facilitators of advance care planning among sexual and gender minority individuals.

## Introduction

More than half of sexual and gender minority (SGM) adults experience some form of discrimination in health care; for those who are transgender or gender nonconforming, the percentage increases to 70%.^1^ SGM communities face multilevel stressors, including family rejection, financial insecurity, and anxiety over concealment of their sexual orientation and/or gender identity; the stress of discrimination toward SGM individuals can reduce life expectancy by up to 12 years.^2^ SGM people have been left out of the recent conversations regarding the outcomes (or lack thereof) of advance care planning (ACP)^3^ because there is little evidence, data, or stories from SGM communities about the value (or not) of ACP.^4^ While ACP may positively affect quality end-of-life (EOL) care and ensure preferences are aligned,^5^ ACP among SGM is not well understood, and patient-centered approaches for this population are lacking.

Existing studies from Canada^6^ and Australia^7^ suggest a need to foster greater engagement in ACP among SGM people, given low ACP rates, historical mistrust, and a lack of training for clinicians to address planning needs unique to this population, including documentation. A review of EOL preparatory behaviors found a low prevalence of ACP discussions between SGM people and primary care clinicians, particularly for transgender people.^8^

Little is known about the ACP experiences and documentation for this group. This study examines readiness of SGM people to engage in ACP and appoint a medical decision-maker (MDM), concerns about discrimination, and their experiences with EOL discussions with MDMs.

## Methods

### Study Design

In this study, a national, explanatory sequential mixed methods design was used.^9,10,11^ In the first phase, we collected survey data, and in the second phase, we conducted qualitative telephone interviews. Four items related to ACP and documentation were inserted into a nationally representative weekly omnibus phone survey conducted by SSRS research over a period of 7 weeks between October and November 2020. The study protocol was approved by the MassGeneralBrigham institutional review board and reporting adheres to Consolidated Criteria for Reporting Qualitative Research (COREQ) ^12^ and American Association for Public Opinion Research (AAPOR)^13^ guidelines.

### Sample

Adults 18 years of age and older were included, with SGM respondents self-identified through a demographic item and a random sample of non-SGM respondents. The survey was designed to include 200 SGM and 400 non-SGM participants to provide sufficient power to compare responses across groups. Furthermore, we estimated that a sample of 175 to 200 SGM participants would yield enough people who would respond yes to a follow-up interview (assuming 66 affirmative responses out of a cohort of 200, with 95% confidence and 10% margin of error). Race and ethnicity data were collected to characterize the sample and assess for differences in ACP. Self-reported categories included Asian; Black Hispanic; Black non-Hispanic; Native American, American Indian, or Alaska Native; White Hispanic; White non-Hispanic; multiracial and other race.

### Survey

The ACP survey items were adapted from the validated survey questions for ACP engagement in the study by Sudore et al,^14^ including an option to capture the role of discrimination in hesitancy to speak about wishes for EOL. We also included an item inviting SGM respondents to provide contact information for follow-up interviews.

In the second phase of the study, we conducted interviews to help explain the survey data and to learn more about the experiences of discrimination among SGM participants. In line with strong mixed-methods techniques, joint displays were used.

### Interviews

The qualitative interview guide was developed after the survey data collection period. Draft domains were based on review of the literature and were informed by the survey results. Domains included ACP discussions with clinicians, expectations, perception of quality and effects of ACP, and satisfaction with discussions; selecting MDMs, sharing preferences, and understanding of their role. The draft guide was revised based on feedback from the study expert advisory panel and members of the SGM community to further explore experiences of discrimination, which was reported at higher rates among SGM survey participants, and whether participants felt that their identified MDMs would be supported. Items were also included to ask to what extent participants felt their SGM identities influenced ACP discussions and experiences and whether they would like sexual orientation and/or gender identity (SOGI) information to be collected by clinicians (eAppendix 3 in the Supplement). The guide was pilot tested for length and clarity.^15^ Survey respondents who consented to a follow-up interview were scheduled within 3 to 4 months following the survey (January to March 2021). Interview participants were provided a $50 honorarium for their time.

### Statistical Analysis

#### Data Analysis

Differences in simple proportions between SGM and non-SGM participants were evaluated using χ^2^ tests in SAS version 9.4 (SAS Institute). *P* < .05 was considered significant, and all tests were 2-tailed.

#### Qualitative Analyses

The research team met to discuss emerging concepts and update the interview guide. A preliminary codebook was developed using the interview guide, and two authors (A.J.R. and S.P.) independently tested it on 2 transcripts. These coded transcripts were reviewed line by line to discuss discrepancies and revised based on discussion with the research team and applied to all other transcripts. Twenty percent of transcripts were double-coded by A.J.R. and S.P. and compared to ensure fidelity to the codebook. To avoid bias, multiple members of the research team reviewed the data separately to gather alternative explanations from each researcher’s positionality: physician, social scientist, and research professionals. The team engaged in a process of thematic analysis^16^ of all coded transcripts and interview memos, guided by interpretive description.^17^

## Results

### Characteristics of Survey Participants

The survey included 201 respondents who self-identified as SGM (101 [50.2%] female; 22 [10.9%] Black, 37 [18.4%] Hispanic, and 140 [69.7%] White individuals), and 402 who did not self-identify as SGM (hereafter referred to as non-SGM; 199 [49.5%] female; 35 [8.7%] Black, 41 [10.2%] Hispanic, and 324 [80.6%] White individuals). Compared with non-SGM participants, SGM were slightly younger, at a mean (SD) of 45.7 (18.6) years, than non-SGM, at a mean (SD) of 53.7 (19.2) years and more likely to be single, never married (55 [27.4%] vs 58 [14.4%]). SGM participants were also slightly more likely to be uninsured (32 [19.0%] vs 37 [13.2%]) and to live in urban areas (116 [57.7%] vs 203 [50.5%]) (Table 1).

**Table 1.** Survey Participant Characteristics

Characteristic	Participants, No. (%)	
	SGM (n = 201)	Non-SGM (n = 402)
Age, mean (SD), y	45.7 (18.6)	53.7 (19.2)
Sex (male/female binary categories as captured by survey)		
Male	100 (49.8)	203 (50.5)
Female	101 (50.2)	199 (49.5)
Hispanic ethnicity	37 (18.4)	41 (10.2)
Race		
Asian	3 (1.5)	3 (0.7)
Black or African American	22 (10.9)	35 (8.7)
Native American, American Indian, or Alaska Native	2 (1.0)	5 (1.2)
Native Hawaiian and other Pacific Islander	4 (2.0)	3 (0.7)
White	140 (69.7)	324 (80.6)
Other	16 (8.0)	14 (3.5)
Multiracial	10 (5.5)	15 (3.7)
Refused to answer	3 (1.5)	3 (0.7)
Marital status		
Single, never married	55 (27.4)	58 (14.4)
Single, living with a partner	30 (14.9)	31 (7.7)
Married	68 (33.8)	233 (58.0)
Separated	10 (5.0)	9 (2.2)
Widowed	15 (7.5)	35 (8.7)
Divorced	19 (9.5)	35 (8.7)
Refused to answer	4 (2.0)	1 (0.2)
Do you have health insurance?^a^		
Yes	136 (81.0)	243 (86.8)
No	32 (19.0)	37 (13.2)
Insurance type		
Somewhere else	1 (0.7)	2 (0.8)
Plan through your or your partner’s employer	83 (61.0)	191 (78.6)
Plan you purchased yourself either from an insurance company or a state or federal marketplace	12 (8.8)	5 (2.1)
Medicare-only	8 (5.9)	18 (7.4)
Medicaid	20 (14.7)	18 (7.4)
Plan through your parents, mother, or father	9 (6.6)	6 (2.5)
Do not know	3 (2.2)	0
Refused to answer	0	3 (1.2)
Education		
Did not graduate high school	21 (10.5)	14 (3.5)
High school graduate or GED	39 (19.4)	95 (23.6)
Some college, no degree	25 (12.4)	74 (18.4)
2- or 4-year degree	80 (39.8)	176 (43.8)
Graduate degree	35 (17.4)	42 (10.5)
Refused to answer	1 (0.5)	1 (0.2)
Urban vs rural		
Urban	116 (57.7)	203 (50.5)
Suburban	34 (16.9)	87 (21.6)
Rural	30 (14.9)	88 (21.9)
No metropolitan status	21 (10.4)	24 (6.0)
Region		
Northeast	29 (14.4)	72 (17.9)
Midwest	44 (21.9)	81 (20.1)
South	74 (36.8)	144 (35.8)
West	54 (26.9)	105 (26.1)

Abbreviation: SGM, sexual and gender minority.

^a^ Not all participants responded to this question.

### Characteristics of Interview Participants

Of the 201 SGM survey participants, 75 agreed to enroll in the qualitative portion of the study, and 25 interviews were completed. Differences between those who did and did not participate in interviews appear in the eTable in the Supplement. Participants self-identified as gay (8 [32.0%]), lesbian (5 [20.0%]), bisexual (7 [28.0%]), pansexual (3 [12.0%]), queer (3 [12.0%]), asexual (1 [4.0%]), and polyamorous (1 [4.0%]). Regarding gender identity, the sample included 2 transgender participants (8.0%), 1 nonbinary participant (4.0%), 20 nontransgender participants (80.0%), and 2 who reported unsure (8.0%). Most reported White race (22 [88.0]%) and had high levels of education (23 [92.0%] had at least some college). Overall, 9 (360%) described themselves as single, 4 (16.0%) single living with a partner, 10 (40.0%) married, and 2 (8.0%) divorced (Table 2). Most described their health as at least fair (84%) (eAppendix 1 in the Supplement). Of the 75 who initially agreed, the study team started with the earliest respondents until saturation was reached. Our goal was to have a diverse sample without focusing exclusively on one sexual or gender minority group; experiences were common across the diverse groups interviewed (eAppendix 4 in the Supplement). With the rigorous coding processes the study team had in place, there was a very strong sense of saturation, meaning no new themes emerged, by the time we reached 25 interviews.

**Table 2.** Interview Participant Characteristics

Characteristic	Participants, No. (%) (n = 25)
Age, mean (SD), y	45.2 (15.7)
Sex at birth	
Male	9 (36.0)
Female	16 (64.0)
Sexual orientation^a^	
Gay	8 (32.0)
Lesbian	5 (20.0)
Bisexual	7 (28.0)
Pansexual	3 (12.0)
Queer	3 (12.0)
Asexual	1 (4.0)
Polyamorous	1 (4.0)
Gender identity	
Transgender	2 (8.0)
Cisgender	20 (80.0)
Nonbinary	1 (4.0)
Unsure	2 (8.0)
Hispanic ethnicity	3 (12.0)
Race	
Black or African American	1 (4.0)
White	22 (88.0)
Multiracial	1 (4.0)
Other	1 (4.0)
Marital status	
Single, never married	9 (36.0)
Single, living with a partner	4 (16.0)
Married	10 (40.0)
Divorced	2 (8.0)
Insurance type	
Private	16 (64.0)
Medicare-only	3 (12.0)
Medicare and private	1 (4.0)
Medicare and Medicaid	1 (4.0)
Medicaid	3 (12.0)
Other	1 (4.0)
Education	
High school graduate or GED	2 (8.0)
Some college	4 (16.0)
2- or 4-year degree	11 (44.0)
Graduate degree	8 (32.0)
Region	
Northeast	5 (20.0)
Midwest	3 (12.0)
South	12 (48.0)
West	5 (20.0)
Interview time, mean (SD), min	33.0 (10.1)

^a^ Percentages may not sum to 100 because participants could select more than 1 option.

### Survey Results

SGM and non-SGM participants did not differ significantly in response to survey questions related to readiness to sign official papers naming an MDM or readiness to talk to an MDM about the kind of care they would want at the end of life (Table 3). In response to the question, “If you haven’t spoken about your wishes, why haven’t you?” SGM participants were significantly more likely to select “I don’t see the need” than non-SGM participants (72 [73.5%] vs 131 [57.2%]; *P* = .005) and “I feel discriminated against” (12 [12.2%] vs 6 [2.6%]; *P* < .001). The response to the discrimination item was explored further in interviews.

**Table 3.** Survey Results

Question	No./total No. (%)		*P* value^a^
	SGM	Non-SGM	
How ready are you to sign official papers naming a medical decision-maker to make decisions for you? Would you say…			
You have never thought about it	78/201 (38.8)	152/402 (37.8)	.34
You are planning to do it	40/201 (19.9)	91/402 (22.6)	
You have already done it	82/201 (40.8)	150/402 (37.3)	
Other	1/201 (0.5)	9/402 (2.2)	
How ready are you to talk to your decision-maker about the kind of medical care you would want if you were very sick or near the end of life?			
You have never thought about it	66/201 (32.8)	146/402 (36.3)	.73
You are planning to do it	41/201 (20.4)	86/402 (21.4)	
You have already done it	90/201 (44.8)	161/402 (40.0)	
Other	4/201 (2.0)	9/402 (2.2)	
How ready are you to talk to your doctor about the kind of medical care you would want if you were very sick or near the end of life?			
You have never thought about it	79/201 (39.3)	182/402 (45.3)	.08
You are planning to do it	44/201 (21.9)	99/402 (24.6)	
You have already done it	73/201 (36.3)	106/402 (26.4)	
Other	5/201 (2.5)	15/402 (3.7)	
If you haven’t spoken about your wishes, why haven’t you?^b^			
Don’t see the need			
No	26/98 (26.5)	98/229 (42.8)	.006
Yes	72/98 (73.5)	131/229 (57.2)	
Too difficult a topic			
No	72/98 (73.5)	161/229 (70.3)	.56
Yes	26/98 (26.5)	68/229 (29.7)	
I feel discriminated against by others			
No	86/98 (87.8)	223/229 (97.4)	<.001
Yes	12/98 (12.2)	6/229 (2.6)	
Other			
No	87/98 (88.8)	190/229 (83.0)	.18
Yes	11/98 (11.2)	39/229 (17.0)	

Abbreviation: SGM, sexual and gender minority.

^a^ *P* values derived from χ^2^ test.

^b^ Overall, there were 327 responses to this question. Some respondents chose more than 1 option, so each response was broken into a yes or no binary.

### Qualitative Interview Themes

Three main themes and 2 subthemes were identified (Table 4). These themes captured the impact of discrimination on ACP encounters and beliefs about the usefulness of ACP documents for future care.

**Table 4.** Themes and Selected Quotes

Theme	Quote (participant)
Theme 1: fear of discrimination limits disclosure of sexual orientation/gender identity and impacts selection of clinicians	“… it’s establishing a good relationship if you trust their opinion or their judgment, if I was seeing a clinician at an affirming place and at a place where I felt like I was respected and understood, I think that is really important, especially when you’re talking about something like death” (12)
	“And depending on the doctor, I’ve had a male older doctor go over my form with me. And when he gets to the part of sexual orientation, he goes like, ‘Huh.’ He makes this noise and made me immediately feel uncomfortable like, ‘You’re kind of judging me.’ And I’ve talked to other friends of mine that feel the same way. So then it does make some people not want to be more forthcoming about what their sexual orientation is because people aren’t as welcoming or as understanding that that doesn't mean promiscuous. It doesn’t mean the same thing. Yeah. I’ve had experience” (22)
Subtheme 1: identity in context	“In a small town, to be honest, it was very awkward at first, because it’s one of those things, you know people know, but you just don’t talk about it kind of thing. So you were even hesitant. And I mean, so you were tempted to put friend on there just because you don’t want people out spreading your business. But at this point, we’ve been together 24 y. If they don’t know, they’ve been living under a rock, so. I mean, when you’re in a really small town, everybody knows” (2)
	“I’ve been sick out of my home state twice. And my wife wasn’t here, and it was COVID. Nobody could visit me. It makes me uncertain and a little insecure, I guess, about that possibly happening, whether or not it’s the law. If you fall sick in the wrong place, the wrong town, or state, I don’t know if that would still happen. But it’s definitely been a concern. It’s definitely been something we’ve talked about” (16)
Theme 2: concerns about whether EOL preferences and appointed MDMs would be supported	“I think that’s where it gets tricky because I was just kind of playing out the scenario in my head that if the need arose where he needed to make a decision, who would he be enabled by? I have no familiarity with the law saying that we have an unofficial domestic partnership since we’ve lived together or I don’t know if it would go up to my parents, and I don’t know–and I don’t know if my parents would respect his wishes if that makes sense” (7)
	“He would be supported by my family. The health care system, I’m not so sure because we are not technically married and we don’t have a domestic partnership” (17)
Subtheme 2: legal status of relationship	“One of the concerns that both of us have—and this is what we have seen from information and stories being written about other couples, where you find a judge—or a family member disputes to it, or there’s money involved. And you find a judge that’s not supportive of same-sex relationships, same-sex marriage, and they throw out a will. They throw out any kind of advance care planning and directive. And the spouse, the partner is totally removed from any decisions any intention that the person may have had” (5)
	“But it was tougher, like I said, before we actually got married. Because before, every form, you have to put his name down specifically, ‘You can discuss my medical history with him. In a case of an emergency, call this person,’ but you still don’t know under the heat of fire if I were in the hospital, would they say, ‘You're not allowed in. You’re not an immediate family member’?” (2)
Theme 3: most discussions occur outside clinical settings	“As it becomes more obvious that they don’t have the capacity to do what they used to do, that they need help and we are trusted helpers. Fortunately, I get along great with my mom and dad. … And my wife and her family all are a good unit. And so we trust each other, and I think that helps a little bit as it becomes more obvious of the sort of deterioration that we all are going to go through unless you’re killed accidentally” (26)

Abbreviations: EOL, end of life; MDM, medical decision-maker.

#### Theme 1: Fear of Discrimination Limits Disclosure of SOGI and Affects Selection of Clinicians

Participants reported some situations in which SGM status is very relevant to care, and relevant to ACP, but wanted to first be assured that the information will be managed in a safe and respectful way. While some described a desire to normalize the process of asking about, for example, partners and preferred pronouns, others worried that they may be discriminated against based on that information or did not want to be defined by it.

Participant 20 described hesitation to disclose SGM status to clinicians. “If the reason they were asking [about SOGI] is their absolute written ethical policy was we ask because we want to accommodate the LGBTQ [lesbian, gay, bisexual, transgender, and queer] community, then the answer is yes. If it were for any other reason, the answer is hell no.”

Some proactively address this by noting SGM status early on, while others opt to avoid disclosing. Participant 25 said, “I guess it’s always on the back of my mind that it could be a problem for someone, and that’s why I tend to proactively say before I see someone new, ‘I'm gay,’ in case it’s a problem.”

In general, SGM people described facing an additional burden in establishing a good clinical relationship that would facilitate quality ACP discussions. As participant 17 explained, “It’s influenced the difficulty I have had in finding a primary care physician that I feel comfortable talking with about certain health concerns.” One participant expressed fear over addressing ACP discussions in the context of their identity as an SGM person, “I am really, really scared. I never thought of those two things [referring to SGM and ACP discussions] at the same time” (participant 23).

#### Subtheme 1: Identity in Context

Participants described multiple identities as important to their experiences of health care and ACP, including race, socioeconomic status, and disability, as well as the regional or cultural context in which their identities are understood. Within the United States and across the health care system, some communities may feel more accepting and safe than others. Discrimination, or fear of it, may occur within multiple layers. Others described how different parts of their personal identities intersect with SGM identities and how that impacted ACP.

Many participants pointed to regional or urban/rural differences. For example, participant 3 stated that “not normative in Oklahoma is interesting because you either learn to be very good at hiding it, or you learn to really, really just not care. That’s not the right word. You definitely care. But acknowledge that other people don’t like you.”

Some participants described how different parts of their personal identities intersect with SGM identities. For example, participant 9 said, “I’m autistic. So I am actually far more likely to get issues with advocacy over being autistic than I am over being miscellaneous flavors of queer. But the way those things intersect is very often relevant.”

#### Theme 2: Concerns About Whether EOL Preferences and Appointed MDMs Would Be Supported

SGM participants described concerns over whether their MDMs or medical preferences for EOL care would be supported, by both their family and by the health care system. Participant 6 explained, “I’ve discussed it with my spouse, and she already said, ‘Yes. If you land up in the hospital with something that’s long-term or terminal or something, I’ll just bring your syringe every couple of weeks and give you your testosterone.’ Because the last thing I want to do is maybe get sick for six months and die not looking like the person I lived my life as. … I think it’s very, very important for clinicians to be able to say, ‘Look, if it could lengthen your life by eight months, would you want us to withhold your hormone therapy or vice versa?’ … I think that they need to ask really detailed questions like that.”

While many expressed that families were supportive of their relationships, not all did. For example, participant 10 stated that “even though my mother treated my daughter as one of our family, because she wasn't my birth child—she was my lesbian partner’s birth child—she was always treated just a little bit differently. And so there’s a rift in my family because of that.”

Furthermore, there was concern about how MDMs would be treated or whether they would be respected by the health care system generally. Participant 20 said, “Oh, yeah, if they tried to go over our wishes and try to go to my family, I would not be happy. … But I guess it is something that you have to have a concern about that they try to get your next of kin. I just hope that those forms I fill out are enough.”

#### Subtheme 2: Legal Status of Relationship With a Partner and Impacts on Clinical Interactions and Documentation

Some who were married described the legal aspect of marriage being important because it meant that nobody could deny someone of the same gender the right to make decisions on their behalf. Others worried that the lack of a legal marriage may mean their partner may not be allowed to make decisions. Participant 7 said, “What I fear is my boyfriend and I aren’t married, so I don’t know if my sexual orientation, with us not being married—whether him being allowed to be a primary decision-maker or him having power of attorney would be more difficult than it would be in a heterosexual couple.”

Another participant described the sense of comfort derived from knowing their relationship had legal status. “Since M is my spouse now, I don’t worry about it as much as before she was my spouse.”

#### Theme 3: Most Discussions About EOL Preferences Occurred Without Clinicians

While relatively few participants described formal ACP in a clinical setting, many had discussed EOL wishes with important others and considered and identified (sometimes officially) MDMs. This finding, that most of these discussions occur outside of clinician settings, is consistent with patterns seen among non-SGM people as well.^18^

Many described detailed discussions with MDMs regarding preferences. For example, participant 5 described their discussion with their spouse: “I know that while quality of life is important to both of us and that is in our plan, if it comes to the point where you’re hooked up on life support and life support is the only thing that's keeping you going, unplug it. …That’s not something that either one of us want, to be kept here, our bodies just kept alive, waiting. So we’ve discussed that.”

### Integrated Mixed Methods Results

Through the use of joint display analysis, we used side by side comparison of the survey and interview data^19^ to assess for confirmation, expansion, or discordance between the data sets (eFigure in the Supplement). Survey results indicate that SGM and non-SGM individuals did not differ significantly regarding naming and talking to MDMs. However, there were important differences in why they have not spoken to their clinician, and challenges with clinical encounters related to being SGM were described in the qualitative interviews (eFigure in the Supplement). Survey and interview data were concordant except for SGM participants citing they “don’t see the need” to speak about wishes if they had not. While 73% of survey respondents who had not spoken about wishes denied the need for these discussions, in interviews that asked why they did not feel the need to document their wishes—regardless of age—participants described concerns about whether preferences would be honored and MDMs would be supported. Younger participants who had spoken about wishes often described more complicated health statuses that motivated documentation. Participants offered recommendations on how to improve the ACP experience among SGM and clinicians. Recommendations focused on taking an honest and direct approach to discussions, promoting inclusion, and avoiding judgment (eAppendix 2 in the Supplement).

## Discussion

This national mixed-methods study found that discrimination and fear of discrimination in clinical settings may be an important factor influencing how SGM people engage in (or avoid) health care and ACP discussions. This extends to relationships with clinicians and comfort having ACP or confidence that personal wishes would be supported. In interviews, participants reported a spectrum of preferences regarding whether they wanted clinicians to ask for SOGI data. Many described clinical encounters in which acceptance, understanding, and support of SGM people was not clearly expressed by clinicians or health care organizations. The important connecting factor was a need to be assured that they would be treated safely and respectfully.

These findings are consistent with literature regarding fear and discrimination preventing SOGI disclosure and delaying entry to care^20^ and suggest that the health care system could make improvements to better support the ACP needs of SGM. Clinicians can take proactive measures to promote inclusivity and signal to patients that they will provide care in a nonjudgmental manner. A study of nurses found a lack of knowledge about advance directives for SGM, difficulties in having conversations, and a need for education and training in ACP for this population.^21^ Demeester et al^22^ offer a conceptual model to promote shared decision-making for SGM and historically marginalized populations, which includes 6 organization-level drivers (workflows, health information technology, organizational structure and culture, resources and clinic environment, education and training, and incentives and disincentives). Contextual changes are designed to establish a safe environment, build trust, and reduce stigma.

Participants in this study described approaches clinicians can implement to establish a supportive environment to engage in ACP, including an honest and direct approach, promoting inclusion, and avoiding judgement. The potential to mitigate anticipated bias can have important impacts. An analysis of transgender and gender nonconforming individuals^23^ found that those with higher levels of confidence that health care professionals will treat them with dignity and respect at EOL had increased odds of perceiving that they were aging successfully.

In addition to assuring the clinical environment has a zero-tolerance policy for discrimination, organizations should have current information and training available on state and federal policies relevant to EOL planning among SGM people.^24^ Findings from this study indicate that SGM individuals have documented MDMs at higher rates than non-SGM individuals, yet still worry about whether their preferences and MDMs will be supported. Health care organizations must go beyond documentation and ensure that patients can be confident that their information is recorded and interpreted appropriately.

### Limitations

This study has limitations. First, all SGM people were included in one group, but the diversity of identities within the SGM umbrella makes it difficult to capture all experiences in depth. The survey instrument also limited the gender item to a male and female binary. Second, among those survey participants who agreed to follow-up interviews, our subsample was predominantly White with high education levels. Those who participated in interviews may not reflect the experiences of other SGM people.

## Conclusions

The results of this study indicate that most SGM participants have either already talked to someone about their EOL wishes or are planning to; in interviews, we found that many EOL discussions occurred outside the clinical setting. Clinicians should acknowledge that many SGM patients have already thought about preferences and determine how MDMs should be involved and included in planning processes using language that gives SGM patients and those who care for them space to answer in a way that fits them and their circumstances.

## Notes

Supplement. **eAppendix 1.** Interview Questionnaire **eAppendix 2.** Participant Recommendations for Clinical ACP Encounters **eTable.** Interview Participant vs Noninterview Participant Comparison **eAppendix 3.** Interview Guide **eFigure.** Joint Display Analysis
