# Supplementary material for: Advance Care Planning Experiences Among Sexual and Gender Minority People
Source: JAMA Netw Open. 2022 Jul 20;5(7):e2222993. doi: 10.1001/jamanetworkopen.2022.22993 (PMC9301514; doi:10.1001/jamanetworkopen.2022.22993)
Supplement: Supplement. — eAppendix 1. Interview Questionnaire eAppendix 2. Participant Recommendations for Clinical ACP Encounters eTable. Interview Participant vs Noninterview Participant Comparison eAppendix 3. Interview Guide eFigure. Joint Display Analysis [file jamanetwopen-e2222993-s001.pdf]

Supplemental Online Content

Reich AJ, Perez S, Fleming J, et al. Advance care planning experiences among sexual and gender minority people. *JAMA Netw Open.* 2022;5(7):e2222993. doi:10.1001/jamanetworkopen.2022.22993

eAppendix 1. Interview Questionnaire

eAppendix 2. Participant Recommendations for Clinical ACP Encounters

eTable. Interview Participant vs Noninterview Participant Comparison

eAppendix 3. Interview Guide

eFigure. Joint Display Analysis

This supplemental material has been provided by the authors to give readers additional information about their work.

© 2022 Reich AJ et al. *JAMA Network Open.*

eAppendix 1 Interview Questionnaire

Patient health care questionnaire	N=25 (%)
**How would you rate your overall health? N, (%)**	
Poor	4 (16.0)
Fair	8 (32.0)
Good	10 (40.0)
Very Good	3 (12.0)
Excellent	0
**Who do you consider to be your main healthcare provider? N, (%)**	
Primary care doctor	16 (64.0)
Specialist	4 (16.0)
PCP and specialist	1 (4.0)
Nurse	1 (4.0)
Someone else	1 (4.0)
I don't have a main healthcare provider.	2 (8.0)
Did not answer	
**How often does your main healthcare provider explain things in a way that is easy to understand? N, (%)**	
Never	1 (4.0)
Sometimes	2 (8.0)
Usually	2 (8.0)
Always	20 (80.0)
**Have you had an ACP discussion with a member of your health care team? N, (%)**	
Yes, with my main health care provider	3 (12.0)
Yes, with another member of my healthcare team	3 (12.0)
Yes, both my main provider and another member of my team	1 (4.0)
No	18 (72.0)

**How satisfied were you, overall, with the ACP discussions(s) you've had with a member of your healthcare team?**	
Very satisfied	2
Somewhat satisfied	3
Somewhat dissatisfied	2
**Which of the following forms have you completed (or were completed for you)? N, (%)**	
Living will: yes, no, I don't know	
Yes	7 (28.0)
No	15 (60.0)
I don't know	1 (4.0)
Did not answer	2 (8.0)
Health care proxy: yes, no, I don't know	
Yes	9 (36.0)
No	12 (48.0)
I don't know	3 (12.0)
Did not answer	1 (4.0)
Advance Directive: yes, no, I don't know	
Yes	8 (32.0)
No	12 (48.0)
I don't know	2 (8.0)
Did not answer	3 (12.0)
MOLST or POLST: yes, no, I don't know	
Yes	1 (4.0)
No	14 (56.0)
I don't know	5 (20.0)
Did not answer	5 (20.0)
**Do you think your main healthcare provider knows the kinds of treatment you would want if you could no longer speak for yourself? N, (%)**	
Probably not	16 (64.0)

© 2022 Reich AJ et al. *JAMA Network Open.*

Possibly yes	4 (16.0)
Probably yes	5 (20.0)

eAppendix 2. Participant Recommendations for Clinical ACP Encounters

Recommendation	Participant quote
Take an honest and direct approach	**(participant 6):** *I think they need to be taught to ask the question straightforward. Making assumptions is very dangerous.*<br>*... And honesty, though, a lot of it comes down to time. They don't have the time, I think, to think about these things, but these are the most important things. How do I address you? How should I see you? How do you see yourself? How do you feel about this? Yeah. How would you want your grandkids to see you? Have you thought about this and that? I think that they don't have the time to do that right now with patients. But it should be something that's addressed. The second you meet, it just should be normalized. What are your pronouns? What was listed on your birth certificate? Does this currently have any impact on your life, or is this just another detail just like the birthmark you have on your right hand since you were born?*
Promote inclusion	**(participant 10)** *Well, I think the most important-- to me, I think that they-- well, I don't know how to put this. I think I just said it, that I want to be assured in the way they're presenting themselves, that they're acknowledging and including my unusual family for what it is. And for God's sakes, don't be phony.*
Avoid judgement	**(participant 25):** *I guess in general I would say, "Take seriously what the person is saying and asking, and don't say, 'Don't worry about that,' or don't say, 'You're not going to feel that way.'"*<br><br>*...It'd be wonderful if a doctor said, "This is a no-judgment zone. Let me know whatever you're thinking, whatever you're feeling. This is very important*

© 2022 Reich AJ et al. *JAMA Network Open.*

eTable. Interview Participant vs Noninterview Participant Comparison

	**Non-interview participant**	**Interview participant**
**Survey Respondents, N**	201	25
**Age, mean (SD)**	45.7 (18.6)	45.2 (15.7)
**Sex, N (%)**<br>(male/female binary categories as captured by survey)		
Male	91 (51.7)	9 (36.0)
Female	85 (48.3)	16 (64.0)
**Hispanic ethnicity, N (%)**	34 (19.3)	3 (12.0)
**Race, N (%)**		
White	118 (67.0)	22 (88.0)
Black or African American	21 (11.9)	1 (4.0)
Asian	3 (1.7)	0
Native American/American Indian/Alaska Native	2 (1.1)	0
Native Hawaiian and other Pacific Islander	4 (2.3)	0
Other	15 (8.5)	1 (4.0)
Mixed	9 (5.1)	1 (4.0)
Refused	3 (1.7)	0
**Marital status, N (%)**		
Single, that is never married	46 (26.1)	9 (36.0)
Single, living with a partner	26 (14.7)	4 (16.0)
Married	58 (31.2)	10 (40.0)
Separated	10 (5.6)	0
Widowed	15 (8.5)	0
Divorced	17 (9.6)	2 (8.0)
Refused	4 (2.2)	0
**Education, N (%)**		
Less than High School, High school graduate or GED	58 (32.9)	2 (8.0)

© 2022 Reich AJ et al. *JAMA Network Open.*

Some college, no degree	21 (11.9)	4 (16.0)
Two- or four-year degree	69 (39.2)	11 (44.0)
Graduate Degree	27 (15.3)	8 (32.0)
refused	1 (0.5)	0
**Region, N (%)**		
Northeast	24 (13.6)	5 (20.0)
Midwest	41 (23.3)	3 (12.0)
South	62 (35.2)	12 (48.0)
West	49 (27.8)	5 (20.0)

© 2022 Reich AJ et al. *JAMA Network Open.*

eAppendix 3. Interview Guide

Interviewee:	
Interviewer(s):	
Date of Interview:	

Start Time
End Time
Location

SGM Interview Guide

Verbal Consent Form

Thank you for agreeing to participate today. We are researchers affiliated with Brigham and Women's Hospital. The goal of this study is to learn about people's experiences with having advance care planning conversations. Advance care planning, or ACP, conversations are discussions that occur between patients, caregivers, and clinicians about preferences for future medical care if your health declines. These conversations include discussing what patients value, their hopes, goals and concerns about the future that could influence the type of medical care they want or don't want. We will use the term 'clinician' to refer to members of the care team, including physicians, social workers, nurses, and chaplains.

We are especially interested in what makes ACP easy or hard for sexual and gender minority (SGM) people. SGM is an umbrella term that includes indiviuals who identify as lesbian, gay, bisexual, transgender, or queer. Our goal is to better understand ACP from the perspective of SGM people and ultimately help to improve the experience.

We would like to hear your views on the ACP conversation experiences that you have had, would like to have, or things getting in the way to having them. We are particularly interested in what makes these conversations hard to have, and what might make them easier.

Your participation is voluntary and you are free to decide whether or not to participate and to leave at any time. Please be assured that all of your responses will be kept confidential-meaning it will not be shared outside of the group with anyone, including your health care team. We will not associate your name or other identifying information with anything you say in the interview. Your participation will not affect ongoing or future care, so be as open and honest as you can. We understand how important it is that this information is kept private and confidential.

Do you have any questions before we begin? If you have further questions regarding this study or the interview questions *now or later*, please feel free to ask a member of the research team or contact the investigator listed below. Thank you for your cooperation.

Joel Weissman, PhD
Deputy Director, Chief Scientific Officer, Center for Surgery and Public Health
Brigham and Women's Hospital; (617) 525-7727; jweissman@partners.org
Amanda Reich, PhD, MPH
Research Scientist
Center for Surgery and Public Health; (617) 525-9287; ajreich@bwh.harvard.edu

© 2022 Reich AJ et al. *JAMA Network Open.*

If you'd like to speak to someone not involved in this research about your rights as a research subject, or any concerns or complaints you may have about the research, contact the Partners Human Research Committee at (857) 282-1900.

Would you be willing to participate in this interview? Yes No

If it is acceptable to you, we would like to audio record our conversation today so that we can make sure to capture your thoughts, opinions, and ideas. This will allow us to perform a more detailed analysis later. Again, we would like to emphasize that we will treat your answers with complete confidentiality.

Would it be okay with you if we audio record this conversation? Yes No

If not, we will take written notes to document the content of the conversation.

Do you have any questions before we begin?

Joel Weissman, PhD (PI) and Amanda Reich, PhD, MPH

© 2022 Reich AJ et al. *JAMA Network Open.*

Demographics/pronouns
Before we begin the interview, we'd like to ask you a few demographic questions.
First, can you please tell me what pronouns you prefer to use? *(I use she/her for myself)*
Pronouns:

1. **Do you think of yourself as:**
   - ☐ **Lesbian or gay**
   - ☐ **Straight, that is, not lesbian or gay**
   - ☐ **Bisexual**
   - ☐ **Not listed above (please specify)**
   - ☐ **Not sure** (find out whether they aren't sure they understand the question, or are unsure of/questioning their sexual orientation)
2. **What was your sex at birth (meaning, on your original birth certificate)?**
   - ☐ **Male**
   - ☐ **Female**

Some people identify as transgender if they have a different gender identity from their sex at birth. For example, a person born into a female body, but who feels male or lives as a man.

3. **Do you consider yourself to be transgender?**
   - ☐ **Yes**
   - ☐ **No**
   - ☐ **Non-binary**
   - ☐ **Not sure** (find out whether they aren't sure they understand the question, or aren't sure of/questioning their gender identity)

4. **Do you have a health care proxy, or someone who would make medical decisions for your care if you are unable to?**

☐ Yes
☐ No
☐ Don't know
☐ Refused

5. (if yes) **Who is your health care proxy?**

6. **Proxy's relationship to the patient**:

© 2022 Reich AJ et al. *JAMA Network Open.*

☐ Spouse

☐ Partner

☐ Son or daughter

☐ Grandson or granddaughter

☐ Sibling

☐ Niece or nephew

☐ Legal guardian

☐ Other _______________________________

7. **What are your proxy's preferred pronouns?**

8. **What is your age?**

9. **Would you like for clinicians to ask for information about your sexual orientation or gender identity?** *(data collection preferences)*

Thank you. Now, we would like to ask you some questions about your experience with the healthcare system and Advance Care Planning, or ACP-which are discussions you might have with your clinician about your preferences for future medical care. Since this is voluntary, if there are questions you don't feel comfortable answering, please just let me know and we will skip.

1. **From time to time, most people discuss important matters with other people. Looking back over the last six months-who are the people with whom you discussed matters that are important to you?**

2. **What role, if any, has your sexual orientation/gender identity influenced your clinical experience and care? (with your regular doctor or primary care)**

    a. **Do you think it's important that your clinicians know your sexual orientation or gender identity?**
    b. **Under what circumstances would you disclose your sexual orientation or gender identity when you go to the doctor?**
    c. **Are your family and friends who might be involved in these discussions aware of your sexual orientation/gender identity?**

© 2022 Reich AJ et al. *JAMA Network Open.*

3. **Have you ever heard of ACP? And if so, what do you know about it?**

4. **Have you ever had an ACP discussion?**
   a. **If yes, can you tell us about it? (probes: who was there? What did they ask you? Was this at a planned visit with your clinician? Did you document any decisions?)**

      i. **Can you describe your interaction with your clinician during ACP?**
      ii. **Did you feel satisfied with this interaction?**
      iii. **Did you feel like your goals and treatment preferences were considered?**
      iv. **If others were present (family, friends), how were they involved?**
      v. **Did you feel like your care meaningfully changed because of the discussion?**
      vi. **To what extent do you feel that your sexual orientation or gender identity influenced this discussion/experience?**

   b. **If not, why not?**
      Probe: What role, if any, has your sexual orientation/gender identity played in your not having an ACP discussion?
      Imagine you were taking a walk and a car ran a stop sign and hit part of your body. You were rushed off to a hospital and the first question the nurse asked when you arrived is: "*who do want to make decisions for you if you no longer become able to make them on your own*?"
      - **Would it be easy to think of someone?**
      - **Who would it be?**

      i. **Would you like to have an ACP discussion? (probes: who would you like to have present for the discussion?)**
         1. (if yes, would like to have ACP) **What are your expectations for an ACP discussion (what do you hope to get out of it?)**
         2. (if do not wish to have one): **why not?**

5. **Have your own preferences for care changed over time? How?** *(stability of preferences)*

6. **What do you think makes these discussions difficult?** *(barriers)*

7. **What do you think makes these discussions easier?** *(facilitators)*

© 2022 Reich AJ et al. *JAMA Network Open.*

a. **If had ACP: if you could make one change to the way your doctor talks to you about ACP, what would it be?**
b. **If NO ACP: If you were to talk about ACP with your doctor, what advice would you give them before meeting with you?**
c. **Have you sought out an affirming provider (or someone supportive of LGBTQ community)?**

Next, we will ask you some questions about choosing a healthcare proxy, or someone to make medical decisions for you if you are unable to.

8. (If proxy indicated above, in demographics): **How did you choose your healthcare proxy?**
   a. **Have you written anything down to name them, such as a Power of Attorney?**
   b. **What is your relationship with this person?**
   c. **Do you feel that this person will be supported in making decisions? (by the healthcare system? Clinicians? Participant's family/friends?)**

9. (if indicated NO proxy above, in demographics): **If no, can you tell us why?**

10. **Can you tell us about any discussions you've had about your medical preferences with your proxy or potential proxy?**
    a. **How often do you revisit these discussions? (PROBE: what happens if your life circumstances change?)** *(stability of preferences)*

11. **What do you think the role of your proxy should be in putting information about your preferences in writing?** *(role of proxy in drafting ADs)*

12. **Is there anything else you want to say or share that you think is important we know?**

eFigure. Joint Display Analysis

**Survey results**	**Meta inferences**	**Theme and exemplar quote**
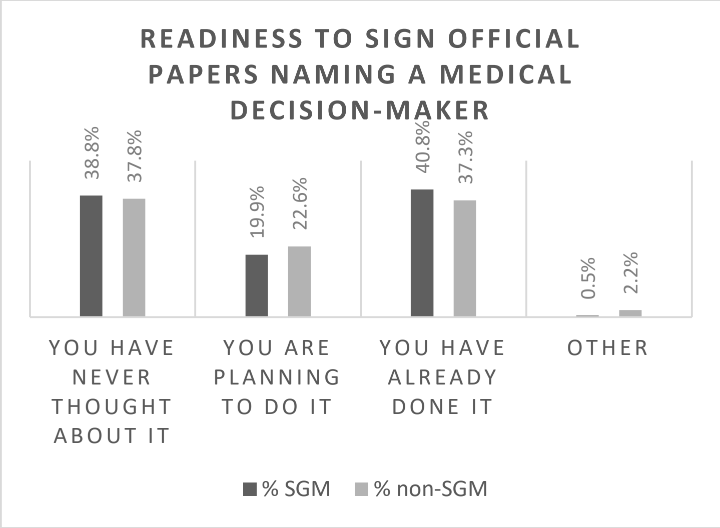	Concordance between survey and interview data. While readiness and rates of documentation of ACP are similar between groups, the interview data further explains how ACP is more complicated among SGM	**Theme: Legal status of relationship**<br>*One of the concerns that both of us have-- and this is what we have seen from information and stories being written about other couples, where you find a judge-- or a family member disputes to it, or there's money involved. And you find a judge that's not supportive of same-sex relationships, same-sex marriage, and they throw out a will. They throw out any kind of advance care planning and directive. And the spouse, the partner is totally removed from any decisions any intention that the person may have had."*<br><br>*But it was tougher, like I said, before we actually got married. Because*

		before, every form, you have to put his name down specifically, "You can discuss my medical history with him. In a case of an emergency, call this person," but you still don't know under the heat of fire if I were in the hospital, would they say, "You're not allowed in. You're not an immediate family member"?
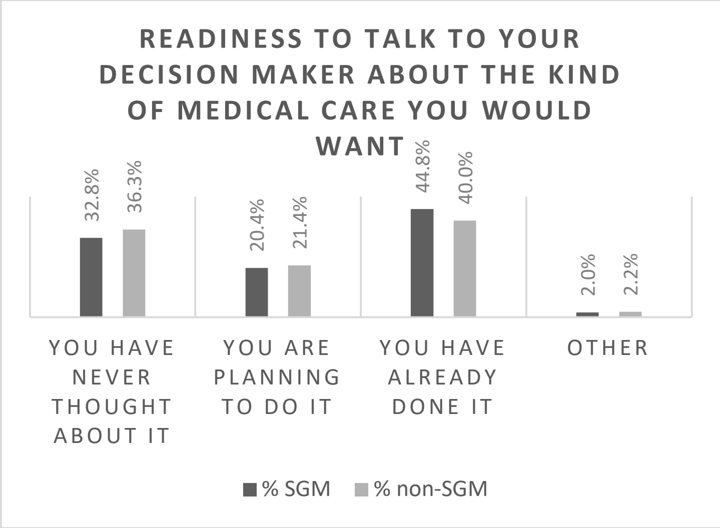	Concordance between survey and interview data.	**Theme: Most discussions occur outside clinical settings**<br>*"But my take is that Mary and my sisters know what I want. And so if they're approached or whatever, they'll know what I prefer." (Participant 18)*<br><br>*"My partner and I discussed it, and we went together and had wills drawn up and the healthcare directives and the powers of attorney and the whole nine yards drawn up." (Participant 2)*

© 2022 Reich AJ et al. *JAMA Network Open.*

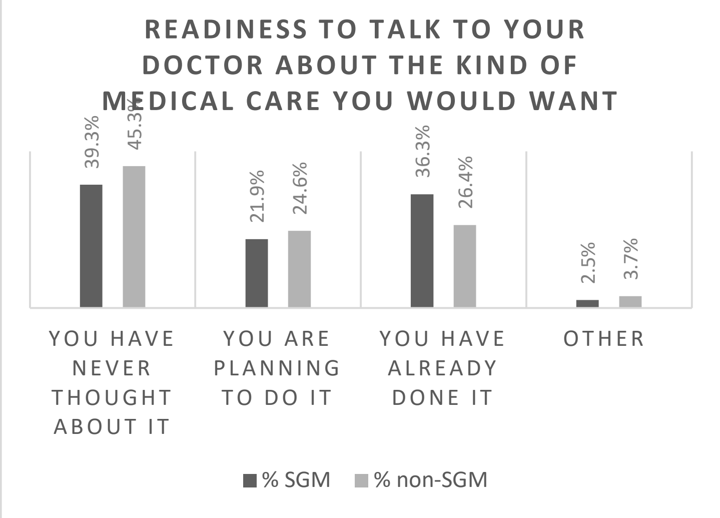	Concordance between survey and interview data and further explains quant data. Qualitative data helps to explain readiness of SGM to discuss ACP and how sexual orientation/gender identity may present a potential barrier to ACP conversations.	**Theme: Fear and experiences of discrimination impact selection of clinicians and whether to disclose sexual orientation/gender identity.**<br>*… it's establishing a good relationship if you trust their opinion or their judgment, if I was seeing a clinician at an affirming place and at a place where I felt like I was respected and understood, I think that is really important, especially when you're talking about something like death*<br><br>*I mean I think if somebody is hiding that they're homosexual, they may also be hiding their partner. So you might open up that whole discussion where now you have a next of kin that is documented. And maybe if you have HIV or something, it might be more important for you to have a next of kin named. (Participant 8)*

© 2022 Reich AJ et al. *JAMA Network Open.*

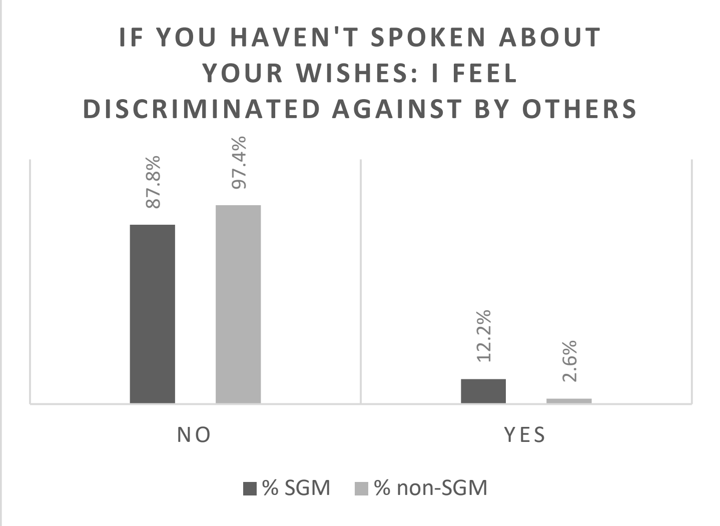	Interview data expands survey results r/t discrimination. Interview data provides examples of discrimination that may be barriers to ACP conversations.	**Theme: Fear and experiences of discrimination impact selection of clinicians and whether to disclose sexual orientation/gender identity**<br>*And depending on the doctor, I've had a male older doctor go over my form with me. And when he gets to the part of sexual orientation, he goes like, "Huh." He makes this noise and made me immediately feel uncomfortable like, "You're kind of judging me." And I've talked to other friends of mine that feel the same way. So then it does make some people not want to be more forthcoming about what their sexual orientation is because people aren't as welcoming or as understanding that that doesn't mean promiscuous. It doesn't mean the same thing. (Participant 22)*
		**Subtheme: Identity in context**<br>*I've been sick out of my home state twice. And my wife*

© 2022 Reich AJ et al. *JAMA Network Open.*

		wasn't here, and it was COVID. Nobody could visit me. It makes me uncertain and a little insecure, I guess, about that possibly happening, whether or not it's the law. If you fall sick in the wrong place, the wrong town, or state, I don't know if that would still happen. But it's definitely been a concern. It's definitely been something we've talked about. (Participant 16)
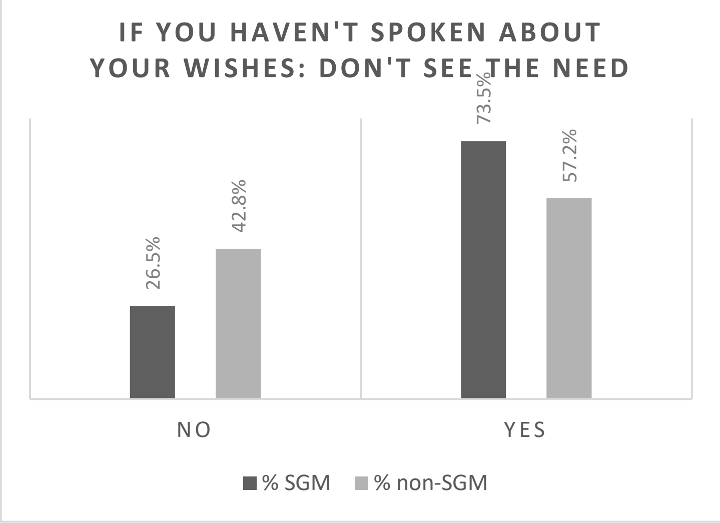	Discordance between interview and survey data. Survey results demonstrate SGM don't see the need for conversations, yet the interviews reveal concern that MDM may not be supported. More needs to be known. Do SGM not see the need because of mistrust that their wishes will be respected.	**Theme: Concerns about whether EOL preferences and appointed medical decision-makers would be supported** *I think that's where it gets tricky because I was just kind of playing out the scenario in my head that if the need arose where he needed to make a decision, who would he be enabled by? I have no familiarity with the law saying that we have an unofficial domestic partnership since we've lived together or I don't*

		know if it would go up to my parents, and I don't know-- and I don't know if my parents would respect his wishes if that makes sense. (Participant 7)<br><br>*He would be supported by my family. The healthcare system, I'm not so sure because we are not technically married and we don't have a domestic partnership. (Participant 17)*

© 2022 Reich AJ et al. *JAMA Network Open.*
